# Ultra-High Dose-Rate Oxygen Depletion and Skin Response to Irradiation

**DOI:** 10.3390/cancers18122011

**Published:** 2026-06-22

**Authors:** Qianyi Huang, Leo Gerweck, Peigen Huang, Ethan Cascio, Bethany Rothwell, Teresa Rodríguez González, Jacob P. Sunnerberg, Megan A. Clark, Jan Schuemann

**Affiliations:** 1 Physics Division, Department of Radiation Oncology, Massachusetts General Hospital and Harvard Medical School, Boston, MA 02114, USA; 2 Edwin L. Steele Laboratory, Department of Radiation Oncology, Massachusetts General Hospital and Harvard Medical School, Boston, MA 02114, USA; 3 Thayer School of Engineering, Dartmouth College, Hanover, NH 03755, USA

**Keywords:** FLASH, FLASH oxygen depletion, normal tissue sparing

## Abstract

**Background/Objectives**: This study investigates the hypothesis that transient oxygen depletion is the mechanism of the skin sparing effect of ultra-high dose-rate irradiation, commonly referred to as FLASH irradiation. **Methods**: Two skin tattoo dots were placed approximately 1.0 cm apart on the thigh of FVB/N mice. The area overlapping the dots was irradiated with a single dose of 27 Gy protons delivered with either FLASH (~120 Gy/s) or 0.5 Gy/s conventional dose-rate (CDR) irradiation. Skin contraction was assessed by measuring the distance between the tattoo dots and complemented by histopathological skin analyses. Mice were placed in a 1.4 L chamber flushed with 5%, 7%, 20.9% or 100% oxygen (balance nitrogen, where applicable) prior to and during irradiation. Skin oxygenation was measured non-invasively using the phosphorescence quenching method. **Results**: Compared to air-breathing mice, skin contraction increases in mice breathing 100% oxygen and decreases when breathing 7% and 5% oxygen following CDR irradiation, showing that skin is neither fully oxygenated nor hypoxic. FLASH irradiation reduced skin contraction, epidermal thickening, and fibrosis in air-breathing mice compared to CDR irradiation. The difference between FLASH and CDR skin contraction decreases as the inspired gas oxygen content is reduced from 20.9% to 7%. Under 5% oxygen breathing conditions, the FLASH sparing effect is eliminated. **Conclusions**: Mean normal tissue pO_2_ does not reveal the presence of cells at low pO_2_ that could become susceptible to FLASH-induced radiobiological hypoxia at doses lower than would be predicted from the mean tissue pO_2_ value. In the absence of oxygen, FLASH skin sparing for the late normal tissue effect, skin contraction, is eliminated.

## Simple Summary

Radiation therapy is a principal form of cancer treatment. However, the dose that can be delivered to tumors is limited by the tolerance of surrounding healthy tissues, such as the heart, lung, and liver. In 2014, it was reported that ultra-high dose-rate irradiation reduced fibrosis in normal lung tissue. Similar findings have since been reported; however, a consensus mechanism underlying normal tissue sparing has not yet been established. In this study, we report that, in contrast to conventional dose-rate irradiation, ultra-high dose-rate irradiation depletes the potent radiation sensitizer, tissue oxygen, more rapidly than it is replenished from adjacent capillaries and reduces mouse skin contraction and fibrosis.

## 1. Introduction

Most cancer patients in the United States are treated by radiation therapy alone or in combination with other treatment modalities [1]. Notable improvements in target dose conformality have been achieved over the past 75 years; however, normal tissue toxicities continue to limit the therapeutic tumor dose. Late toxicities (occurring months to years post-treatment in patients) commonly limit the total dose of radiotherapy, as these effects are often permanent, progressive, and severe (e.g., fibrosis, organ dysfunction [2,3]). Early (acute) reactions (e.g., skin/mucosa) occurring during treatment are usually reversible and manageable [3,4]. In this study, we focus on the relationship between tissue oxygenation and the FLASH sparing of skin contraction, a late effect in skin.

In 2014, Favaudon et al. reported that irradiation at dose rates greater than 40 Gy/s spared normal lung tissue compared to conventional dose-rate irradiation (CDR), typically in the range of 0.05–0.5 Gy/s, but was as effective against tumors as CDR irradiation [5]. This paradigm-shifting report has given rise to an expanding number of papers reporting FLASH tissue sparing and proposing potential mechanisms of normal tissue sparing, reviewed in [6,7,8,9]. In spite of the number of papers reporting a FLASH sparing effect, the mechanism of the FLASH normal tissue sparing effect remains the subject of debate [6,7,8,9]. The absence of a settled mechanism or explanation for the FLASH effect hinders its introduction into the clinic. Nevertheless, at least three clinical trials are currently in progress [9]: a randomized phase II selection trial of FLASH and conventional radiotherapy for patients with localized cutaneous squamous cell carcinoma or basal cell carcinoma, a single-arm FLASH treatment of symptomatic bone metastases to thorax with the assessment of pain relief and safety, and a cutaneous melanoma metastases efficacy and toxicity trial.

A frequently proposed explanation of the damage sparing effect of FLASH irradiation is the radiation-induced depletion of oxygen, a powerful enhancer of radiation damage. When exposed to radiation, the water radiolysis radical •OH extracts one hydrogen from intracellular biomolecules, including DNA. Oxygen oxidizes the resulting DNA• radical, yielding the resultant peroxyl DNA-OO•, which is difficult to repair and leads to DNA strand breaks. At low oxygen concentration and in the absence of oxygen, a competing reaction, the sulfhydryl donation of hydrogen to DNAOH•, precludes the oxygen fixation of DNA damage, allowing the repair of the DNA strand break and increased radiation resistance [10,11]. In the absence of oxygen, cells and tissues are ~3 times more resistant to electron, photon and proton irradiation than in its presence. The oxygen concentration dependent 1 to 3-fold change in radiation sensitivity is called the Oxygen Enhancement Ratio (OER). In oxygenated cells and tissues, a loss of oxygen sensitization may occur if oxygen is depleted more rapidly than it is supplied by the nearest capillary, which may happen during FLASH irradiation. Interestingly, in acute skin studies with high-LET low-OER carbon, increased collagen deposition was observed following CDR but not FLASH irradiation, but acute skin reaction did not differ between FLASH and CDR irradiation [12]. In contrast, in studies with low LET entry carbon irradiation, a significant FLASH sparing effect was observed at all evaluated doses, with a dose modifying factor of ~1.5 [12,13].

A dose of radiation sufficiently large to deplete mean human normal tissue pO_2_ [14,15] substantially exceeds clinically relevant radiation doses and the dose (e.g., 10 Gy) at which the FLASH effect has been reported [16,17,18]. For this reason, oxygen depletion is commonly dismissed as a mechanism of the FLASH normal tissue sparing effect [19,20,21,22]. However, median or average tissue pO_2_ values do not reveal the range of pO_2_ values above and below the mean value [14,15] or the change in pO_2_ with increasing distance from oxygen-supplying capillaries. In an analysis considering the impact of the full spectrum of pO_2_ values measured in normal human tissue, including those below the mean value, Zhu et al. reported that oxygen depletion could lead to a FLASH sparing effect in the context of stereotactic body radiotherapy [23]. Prior studies have demonstrated the FLASH sparing of radiation-induced acute skin damage [24,25,26]. Two recent publications evaluated the dose modifying factor of FLASH vs. CDR irradiation for acute skin damage under air- and oxygen-breathing conditions in skin and gut [27], and under air-breathing and tourniquet hypoxic conditions in skin [28].

The present study was designed to test the hypothesis about the relationship between FLASH oxygen depletion and FLASH tissue sparing. Furthermore, we hypothesize that the distribution of cellular oxygen tensions, rather than the mean tissue pO_2_, is the biologically relevant parameter governing the FLASH tissue sparing effect. The response metric is late normal tissue damage, i.e., persisting skin contraction after 25 days. The underlying basis for the design of the study and interpretation of the results of the study reported here are based on the established relationship between oxygen concentration and radiation sensitivity. Oxygen is a strong modifier of radiation sensitivity in the range of 0–15 mmHg (pO_2_) and especially over the range of 0–5 mmHg. Half-maximal radiosensitization occurs at approximately 3.5 mmHg, whereas near-maximal and relatively invariant radiosensitization is observed over the oxygen concentration range of approximately 15–20 mmHg, up to 760 mmHg (100% oxygen at atmospheric pressure). This relationship has been demonstrated and validated in viruses, bacteria, yeast, and mammalian cells, including human tissue-derived cells [29].

## 2. Materials and Methods

### 2.1. Animals

A total of 284 ten- to twelve-week-old male and female FVB/N mice, bred and raised in our defined flora colony, were used in the experiments. Upon entry into experiments, all animals were housed and maintained in micro-isolator cages. Mice were provided standard laboratory chow and acidified water ad libitum. All animal care and procedures were performed in accordance with the Public Health Service Policy on Humane Care of Laboratory Animals and approved by the IACUC (Institutional Animal Care and Use Committee) at MGH.

### 2.2. Beamline and Dosimetry

Proton irradiation was performed at the experimental beamline in the Burr Proton Therapy Center, which delivers a 230 MeV proton beam. The beam was optimized to deliver ultra-high dose-rate protons to a 1.6 × 1.2 cm field [30]. The range of the average dose rates was between 100 and 130 Gy/s for FLASH irradiations and around 0.5 Gy/s for CDR irradiations. Protons were delivered by a C230 isochronous cyclotron (Ion Beam Applications SA, Louvain-la-Neuve, Belgium) with an energy of 228.9 MeV and a radiofrequency of 106 MHz, resulting in bunches of 3 ns with a spacing between the start of each bunch of 9.4 ns. The dose rate was adjusted by varying the beam current at the cyclotron exit by modulating the arc current in the ion source and thus controlling the number of protons available for acceleration. For FLASH irradiations, the cyclotron output current was set to the maximum allowed of 300 nA. For CDR irradiations, a cyclotron exit current of 1.2 nA produced a dose rate of 0.5 Gy/s using the same scattering configuration [31]. Additional details of the beam characteristics and dosimetry can be found in Zhang et al. [32]. Before each irradiation, the beam flatness was optimized using a custom-made 2D scanner [31]. The mice were then placed in the entry plateau region of the beam. Additional details are provided in Supplementary Figure S1.

### 2.3. Response Metric and Irradiation

For the assessment of skin response to irradiation, two tattoo dots (ATS-3 General Rodent Tattoo System, AIMS, Hornell, NY, USA) were placed approximately 1 cm apart on the shaved right rear leg of the mice 1 day prior to irradiation. Following anesthetization with a mix of ketamine (80–100 mg/kg, Zoetis, Parsippany, NJ, USA) and xylazine (10 mg/kg, AnaSed®, Dechra Veterinary Products, Elwood, KS, USA), the mice were placed in a custom plastic mouse holder. The tattooed leg was extended through a baffle and secured by taping the foot to the holder, which was then put into a chamber with an inner volume of 1.4 L. The chamber was flushed with 5–10 L/min gas containing 100%, 7%, or 5% oxygen (balance nitrogen), or room air (20.9% oxygen). To allow for the stabilization of oxygen in the tissue, the mice were kept in the chamber for approximately three minutes before irradiation [33]. Prior to and following irradiation, the distance between the dots was measured at 4-day intervals for 24 days and weekly thereafter for up to 45 days post-irradiation. In addition to varying skin oxygen concentration by varying the oxygen content in the inspired gas, partial results of a prior study (Supplementary Figure S2) are included [34]. The mouse strain, age, response metric, radiation dose rates and beam characteristics are the same as used in the current study. Following anesthetization with ketamine–xylazine, hypoxia was achieved by string ligature. The tattoo-bearing leg was FLASH or CDR irradiated to a dose of 27 or 45 Gy for 3 min following leg tourniquet ligation. The ligature was removed immediately following irradiation.

### 2.4. Evaluation of Oxygen Status

For the evaluation of tissue oxygenation and changes in oxygenation, we used the phosphorescence method, as described by Cao et al., utilizing the phosphorescent probe Oxyphor PdG4 (generously provided by Dr. Brian Pogue’s laboratory, Dartmouth College, Hanover, NH, USA) [22]. Mice legs were subcutaneously injected with 100 μL of a 20 μM concentration of Oxyphor PdG4 ~1 h before irradiation. Oxyphor is used to identify change in pO_2_ but, due to the injection creating a bolus of Oxyphor under the skin, may not be identical to or resolve the relative concentration of oxygen in skin dermis and epidermis, which is mildly hypoxic in both mouse and human skin [35,36], or the fraction of cells at a particular pO_2_. Approximately 1 h after PdG4 injection, mice were placed in a custom plastic mouse holder, which was then placed in the 1.4 L chamber and was flushed with gas at a flow rate of 5–10 L/minute. The pO_2_ was recorded after placing mice in the chamber, and the leg was irradiated with 27 Gy of CDR and FLASH dose rates when the pO_2_ reached a plateau, i.e., within 3 min. Strong scintillation from the optical fibers during FLASH prevented reliable measurement during irradiation, resulting in a ~0.45 s signal gap and a small underestimation of the minimum pO_2_ during FLASH. This underestimation has been estimated to be <1 mmHg [22]. Following irradiation, tissue pO_2_ was continually monitored for up to an additional 5 min. Separate groups of mice of the same age and gender, anesthesia regimen, mouse restraint, and radiation protocol (dose and dose rates) were used for the evaluation of tissue oxygenation and long-term skin contraction following FLASH or CDR irradiation. Tissue pO_2_ was not evaluated in ligature-induced hypoxia. The evidence of metabolic hypoxia was manifest by the rapid change in the skin color of FVB/N mice within 1.0–1.5 min of ligation. Ando et al. examined mouse skin contraction for up to a year following irradiation. The magnitude of low-LET irradiation contraction was dose-dependent throughout the observation period [37].

### 2.5. Histology

Six mice per treatment group were sacrificed at the completion of the irradiation experiments for Hematoxylin and Eosin (H&E) and Masson’s trichrome staining. Skin tissue between the two tattoo dots was fixed in 4% paraformaldehyde and subsequently transferred to the Dana Faber/Harvard Cancer Center Specialized Histopathology Services Core (MGH site) for further processing. H&E staining was performed to assess morphological changes, particularly epidermal thickness, while Masson’s trichrome staining was used to evaluate dermal collagen deposition. Furthermore, six mice from each treatment group were sacrificed at 4, 12, and 18 days post-irradiation for Ki-67 immunostaining to evaluate cellular proliferation in the skin. Images of skin sections were acquired at 200× magnification using an Axio Scan.Z1, (Carl Zeiss Microscopy GmbH, Jena, Germany). Epidermal thickness was determined in a blinded manner by calculating the mean length of five evenly spaced lines drawn perpendicular to the skin surface along the H&E-stained sections. Collagen deposition was quantified in Masson’s trichrome-stained sections by measuring the positively stained area relative to a predefined region of interest (ROI). Histological slides were digitally scanned and analyzed using QuPath (version 0.5.1) [38]. Collagen deposition was quantified in Masson’s trichrome-stained sections by measuring the positively stained area relative to a defined region of interest (ROI). ROIs were manually selected from comparable irradiated skin regions while excluding artifacts and non-tissue areas. Positive collagen staining was identified using a pixel-classification algorithm trained on representative manually annotated images containing both positive and negative staining regions. Multiple representative sections from different treatment groups were included during classifier training to account for variability in staining intensity and tissue morphology. The same trained classifier and analysis parameters were applied uniformly to all samples. For each mouse, collagen-positive area (%) was calculated as the ratio of positively stained pixels to total pixels within the ROI. To minimize variability, ROIs were selected using consistent anatomical landmarks and comparable tissue depths across all samples. Proliferative cells were quantified in Ki-67-stained sections by measuring the percentage of Ki-67-positive cells relative to the total number of cells within the ROI. Ki-67-positive nuclei were identified using a classifier trained on representative annotated images based on staining intensity and nuclear morphology. Identical classifier settings were applied across all samples. For each mouse, the Ki-67-positive cell fraction (%) was defined as the ratio of positive cells to total cells within the ROI. To minimize operator bias, identical ROI selection criteria, classifier settings, and analysis pipelines were used across all treatment groups.

### 2.6. Estimation of Cell Survival from Spectral pO_2_ Distributions

To estimate the impact of heterogeneous tissue oxygenation on radiation response, hypothetical cellular pO_2_ distributions were constructed that were consistent with the experimentally measured mean tissue pO_2_. These distributions were then incorporated into an oxygen-dependent cell survival model following the approach described by Zhu et al. for modeling oxygen depletion effects during irradiation [23]. Here, an iterative approach was used to determine cell survival after irradiation, starting from initial oxygen concentration, delivering a small portion of the dose, adjusting the oxygen concentration for depletion, and delivering the next dose portion until the final delivered dose was reached.

Oxygen depletion during irradiation was described using a G value of 0.38 mmHg/Gy, a half-maximal oxygen sensitization constant of 3.5 mmHg, a maximum OER value of 3, and the dose to reduce the surviving fraction to e^−1^ (under air conditions) of 1.49 Gy based on preliminary data from an in vitro keratinocyte survival curve. Note, OER values are survival curve model-independent. Oxygen depletion per unit dose (the G value) is determined under known and uniform oxygen conditions as the radiation dose required to render initially oxygenated, radiosensitive cells to a state of maximum radioresistance, where maximum resistance corresponds to the sensitivity of cells irradiated under nitrogen conditions. The required dose is directly dependent on the initial oxygen concentration [39,40,41].

At doses greater than 10 Gy, the LQL model transitions to the **hit-target** model. Since the present study evaluates responses at doses up to 27 Gy, the cell surviving fraction (SF) was calculated using the **single-target/single-hit** model:

$$SF=e^{-D/D_{0}},$$

where *D* is the delivered dose and *D*_0_ is the dose required to reduce the surviving fraction to e^−1^. The *D*_0_ value was adjusted in accordance with the OER-dependent change in radiosensitivity resulting from oxygen depletion during irradiation. This procedure was repeated for each initial cellular pO_2_ value within the modeled pO_2_ distributions.

The cumulative surviving fraction was then calculated by summing the contributions from each cellular subpopulation of the initial pO_2_ distribution.

### 2.7. Statistical Analysis

Ten mice were entered into each unique treatment group, i.e., for each combination of oxygen status and dose rate. For the assessment of long-term skin contraction, the distance between the tattoo dots was measured for 45 days post-irradiation. The contraction fraction for FLASH and CDR irradiations (CF_FLASH_ and CF_CDR_) is defined as (the distance at day 0 minus the distance at day *i*)/(Distance at day 0), where day “0” is measured 1 day prior to irradiation and day “*i*” is the measurement day after irradiation. Mean contraction fraction values were calculated as the average of the time points between 30 and 45 days post-irradiation. As the absolute values of the skin contraction for each unique dose rate and breathed oxygen concentration were not identical from experiment to experiment, only studies that included both FLASH and CDR contraction fractions were employed for analysis. For the estimation of the FLASH sparing factors (SFs), the ratios of the CF_CDR_/CF_FLASH_ and their confidence interval were approximated by the Delta method [42]: $SE_{SF}=\frac{CF_{CDR}}{CF_{FLASH}}\times \surd \left({\left(\frac{SE_{CDR}}{CF_{CDR}}\right)}^{2}+{\left(\frac{SE_{FLASH}}{CF_{FLASH}}\right)}^{2}\right)$, where CF_CDR_ and CF_FLASH_ represent the mean contraction fractions under CDR and FLASH conditions, while SE_CDR_ or SE_FLASH_ denote their standard errors. The statistical significance was calculated by the unpaired two-tailed *t-test* or one-way ANOVA via GraphPad Prism (version 10). The significance of differences in the magnitude of the sparing factors was evaluated by the unpaired two-tailed *t-test*. Significance was defined as * *p* < 0.05; ** *p* < 0.01; *** *p* < 0.001.

## 3. Results

### 3.1. FLASH but Not CDR Irradiation Rapidly Depletes Tissue Oxygen

**Figure 1:**
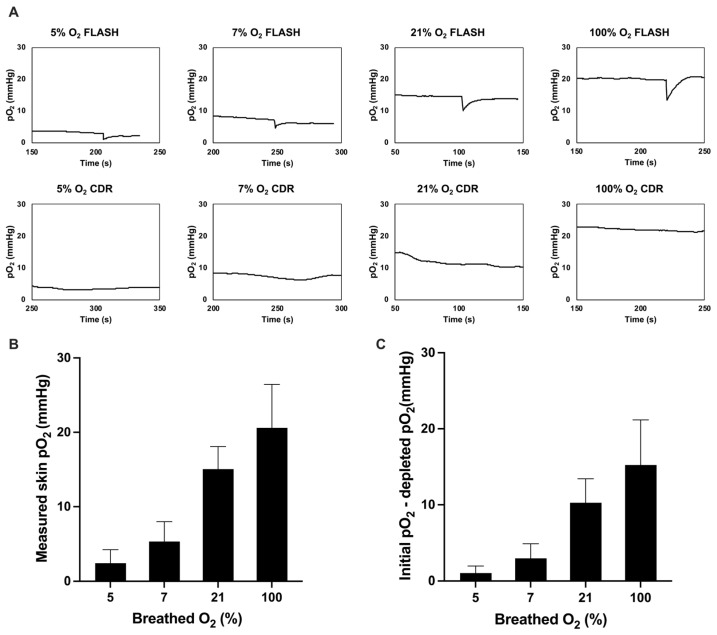
Skin pO_2_ in mice treated with 27 Gy irradiation while breathing 5%, 7%, 21%, and 100% oxygen. (**A**) Skin pO_2_ of representative mice breathing 5%, 7%, 21%, and 100% oxygen following 27 Gy FLASH (**top row**) or CDR (**bottom row**) irradiation. Quantification of (**B**) initial pO_2_ and (**C**) initial pO_2_ minus depleted pO_2_ immediately following FLASH irradiation in mice breathing 5%, 7%, 21%, and 100% oxygen. The initial pO_2_ was the average pO_2_ for approximately 5 s prior to the rapid pO_2_ decrease associated with FLASH irradiation. *n* = 47. Data are presented as Mean ± SD.

As seen in Figure 1, mean tissue pO_2_ strongly correlates with the percent oxygen (balance nitrogen) in the inhaled gas, increasing from approximately 15 mmHg in air-breathing mice to 22 mmHg in mice breathing 100% oxygen and decreasing to 4 mmHg in mice breathing 5% oxygen (Figure 1B). The variability in tissue pO_2_ between mice, as indicated by the confidence intervals, is likely due to both the mouse-to-mouse variability in pO_2_ and variability in the precise injection site of the oxygen probe. The rapid change in tissue pO_2_ status in mice breathing gas containing different oxygen concentrations is consistent with polarographically evaluated skin pO_2_ changes [33]. Differences in dermal and epidermal skin pO_2_ [36] are not resolved in these measurements.

Rapid FLASH oxygen depletion and the recovery from oxygen depletion is seen in Figure 1A. This figure shows that the quantity of FLASH-depleted oxygen depends on the pre-irradiation tissue oxygen concentration. In mice breathing 5% oxygen, there is substantially less depletable and depleted oxygen than in mice breathing gas containing more oxygen. Figure 1A also shows a rapid decrease in oxygenation during FLASH irradiations. There is no depletion observed during CDR irradiation. The average tissue pO_2_ remaining after FLASH irradiations is depicted in Figure 1C. Of note, the phosphorescence emission signal arises from approximately 100–150 mm^3^ of tissue [22], which does not resolve any potential heterogeneities in oxygenation and the percent of tissue at a specific pO_2_ value.

### 3.2. Skin Contraction Is Oxygen-Dependent, Increased at High Tissue pO_2_ and Reduced at Low pO_2_

**Figure 2:**
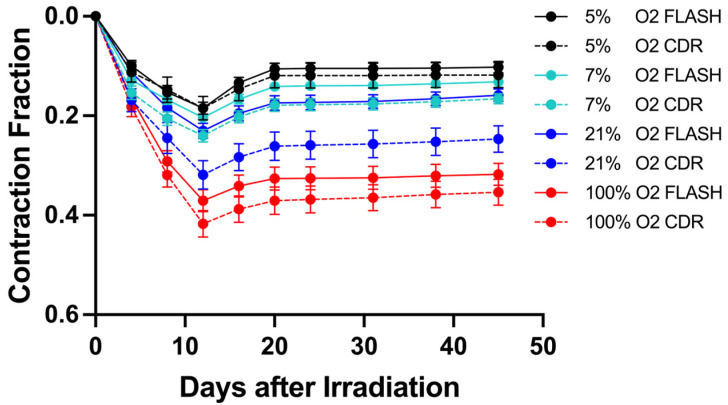
Skin contraction as a function of time following 27 Gy irradiation at FLASH and CDR dose rates in mice breathing 5%, 7%, 21%, and 100% oxygen. Solid curves are for FLASH irradiations, dashed curves are CDR irradiations. *n* = 165, data are presented as Mean ± SEM.

Figure 2 shows the skin contraction fraction in mice receiving 27 Gy CDR or FLASH irradiation while breathing 5% to 100% oxygen. For all oxygen concentrations, acute skin contraction was maximal approximately 12 days post-irradiation, followed by a recovery period and stabilization around day 25. For both CDR and FLASH irradiation, skin contraction increases with increasing tissue oxygen concentration. The most pronounced contraction is seen in CDR irradiated mice breathing 100% oxygen (red dashed curve). Minimal skin contraction is observed in mice breathing 5% oxygen, irrespective of dose rate (black dashed and solid curves). Differences between the FLASH and CDR contraction fractions did not change from day 25 through day 30–45 post-irradiation. Changes in skin contraction following various-sized doses of low-LET irradiation for up to a year post-irradiation reveal a slow gradual increase in contraction [37]. Skin contraction and histology experiments were therefore terminated between day 45 and 60 post-irradiation.

### 3.3. Dependence of FLASH Skin Sparing on Tissue Oxygenation and Hypoxic Conditions

**Figure 3:**
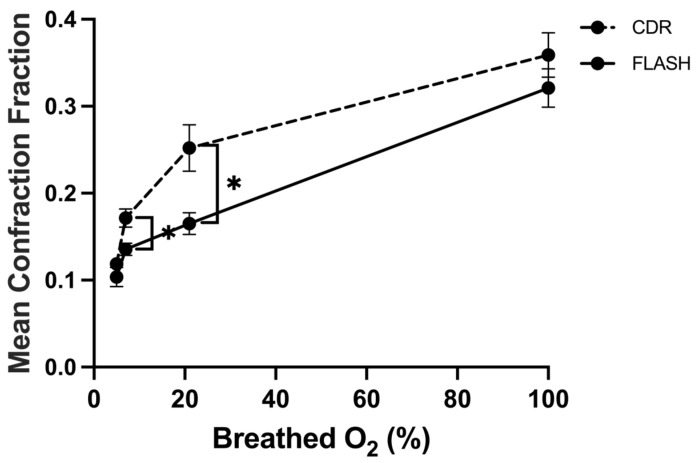
Mean skin contraction values in mice breathing 5–100% oxygen at 30–45 days following a single dose of 27 Gy irradiation. Solid curves are for FLASH irradiations; dashed curves are CDR irradiations. *n* = 165, statistical analysis by unpaired two-tailed *t*-test, * *p* < 0.05. Data are presented as Mean ± SEM.

Figure 3 more clearly illustrates the relationship between oxygen concentration, skin contraction, and the FLASH effect. Skin contraction systematically increases with increasing inspired gas oxygen concentration, regardless of dose rate. At low oxygen concentrations, contraction is significantly reduced, as is the relative magnitude of the FLASH effect. In mice breathing 5% oxygen, a sparing effect due to FLASH irradiation is not observed (*p* = 0.26). FLASH sparing is most pronounced in mice breathing air (20.9% oxygen). The ratio of CDR to FLASH skin contraction decreased from approximately 1.5 under room-air breathing conditions, to approximately 1.25 under 7% oxygen breathing, and to approximately 1.0 under 5% oxygen breathing. FLASH sparing is most pronounced in mice breathing air (20.9% oxygen). In mice breathing 100% oxygen, FLASH sparing is not statistically significant (*p* = 0.27).

**Figure 4:**
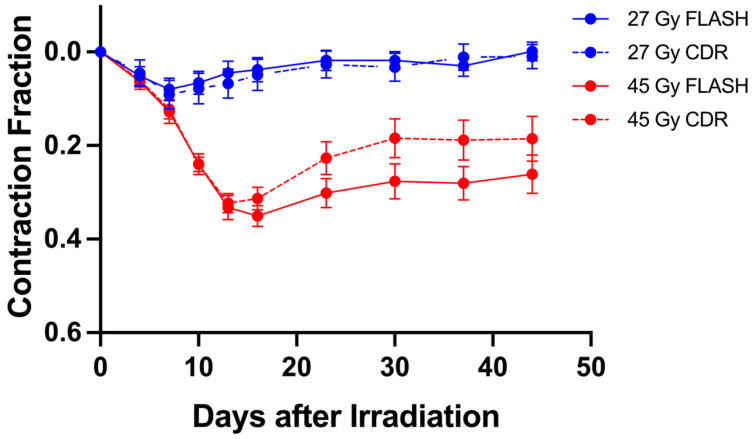
Skin contraction following 27 and 45 Gy under tourniquet hypoxic conditions. Solid curves are for FLASH irradiations; dashed curves are CDR irradiations. *n* = 40, data are presented as Mean ± SEM.

Figure 3 shows that 27 Gy induces significant but minimal contraction in 5% oxygen-breathing mice. Figure 4, which is reproduced from our previous study [34], compares the CDR vs. FLASH effect at 27 Gy and 45 Gy, which induces significantly more skin contraction than 27 Gy. The response of skin irradiated with 27 Gy and 45 Gy that was rendered hypoxic by tourniquet ligation 3 min prior to and during irradiation. Minimal contraction and no FLASH sparing were observed in mice following 27 Gy irradiation, consistent with what was observed following 5% oxygen breathing. A dose of 45 Gy gives rise to significant skin contraction following FLASH and CDR irradiation, but no FLASH protection was observed. Rather, there is a non-significant tendency toward greater contraction following FLASH irradiation (*p* > 0.05).

### 3.4. Histologic Changes Parallel the Reduction in Skin Contraction by FLASH vs. CDR Irradiation

**Figure 5:**
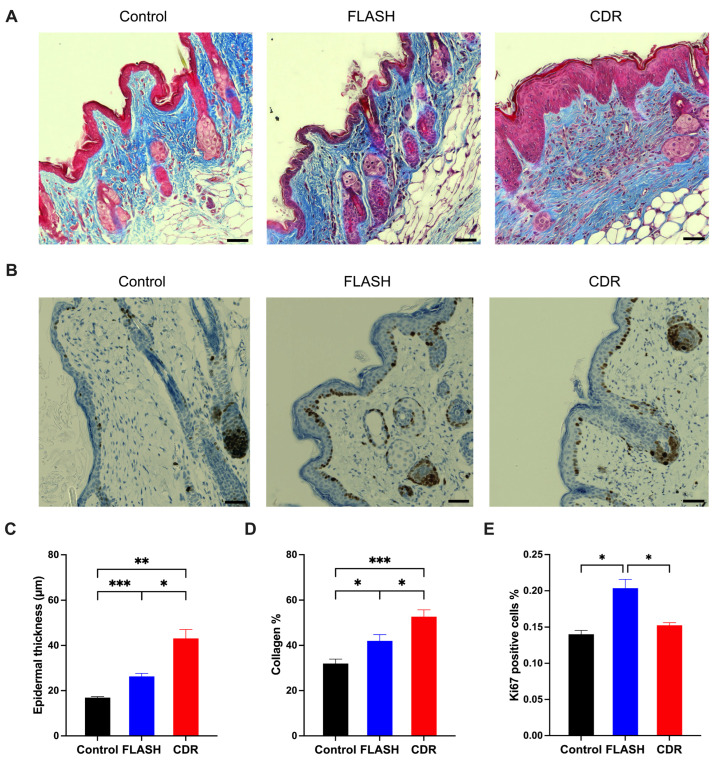
Histologic alterations in mice receiving 27 Gy FLASH or CDR irradiation. Representative images of Masson’s trichrome- (**A**) or Ki67- (**B**) stained skin tissue in mice breathing room air for the unirradiated control or irradiated with 27 Gy at FLASH vs. CDR dose rates, scale bar = 50 µm. Collagen is stained blue in the Masson’s trichrome sections, and Ki-67-positive nuclei are stained black in the Ki67 sections. Quantification of epidermal thickness (**C**), and collagen deposition (**D**) and proliferation (**E**). *n* = 6 for all panels, statistical analysis by one-way ANOVA, * *p* < 0.05; ** *p* < 0.01; *** *p* < 0.001. Data are presented as Mean ± SEM.

Radiation-induced histologic skin changes are shown in Figure 5. Both FLASH and CDR irradiation significantly increased epidermal thickness and dermal collagen density after 60 days following irradiation; however, FLASH irradiation significantly reduced the magnitude of the tissue changes (*p* = 0.02 and 0.03, for epidermal thickness and collagen deposition, respectively). Ki-67 immunostaining was performed to assess cellular proliferation at early time points after irradiation (Supplementary Figure S2). The proliferative activity of epidermal progenitor cells is markedly suppressed on day 4 but increases substantially by day 12 and remained elevated on day 18 for both irradiation modalities. Notably, FLASH irradiation preserves a higher fraction of actively proliferating cells compared with CDR. These proliferating cells are predominantly localized within the basal layer of the epidermis. The impact of altered tissue pO_2_ on histologically evident skin changes is also shown in Supplementary Figure S2. Breathing 5% oxygen significantly preserved epidermal thickness compared with breathing air following either FLASH (*p* = 0.002) or CDR irradiation (*p* = 0.002), with no significant difference observed between FLASH and CDR under hypoxic breathing conditions (*p* = 0.511). In contrast, breathing 100% oxygen significantly increased epidermal thickness following both FLASH (*p* = 0.011) and CDR irradiation (*p* = 0.019), and reduced proliferative activity after both FLASH (*p* = 0.005) and CDR irradiation (*p* = 0.026).

### 3.5. Impact of Oxygen Heterogeneity

**Figure 6:**
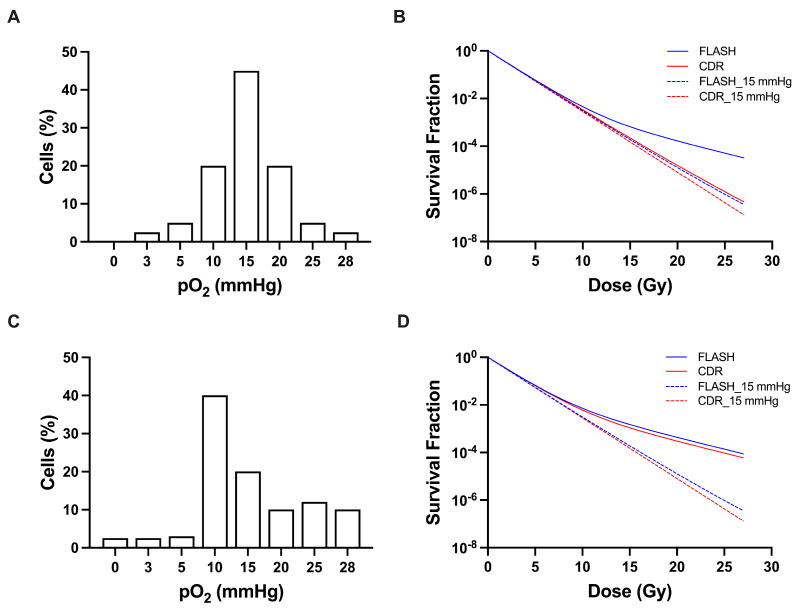
Profiles of skin oxygenation (**A**,**C**) and corresponding effects of CDR and FLASH irradiation (**B**,**D**). Solid curves represent cell survival based on the full spectrum of cell pO_2_ values shown in panels (**A**,**C**), whereas dashed curves represent survival at the measured mean pO_2_ value of 15 mmHg following FLASH (blue) or CDR (red) irradiation.

Figure 6 demonstrates the impact of tissue pO_2_ profiles vs. average tissue pO_2_ on the response to FLASH vs. CDR irradiation. Figure 6A,C shows two hypothetical pO_2_ frequency distributions consistent with the measured mean tissue pO_2_ of 15 mmHg. The distributions were constructed to satisfy the experimentally determined requirement that cells are present both above and below the mean pO_2_ value, with equal cumulative oxygen content (pO_2_ × frequency) on either side of the mean. The impact of 27 Gy irradiation on the survival of cells in the pO_2_ environment in Figure 6A is shown in Figure 6B. The survival of cells in the cell frequency distribution shown in Figure 6C is shown in Figure 6D. The response curves shown in Figure 6B,D were generated using the oxygen-dependent survival model described in the Methods section. These two pO_2_ distributions shown in Figure 6A,C illustrate how different cellular oxygen distributions can yield the same mean tissue pO_2_ while producing markedly different predicted responses to FLASH and CDR irradiation.

The presence of pO_2_ values above and below the mean pO_2_ are based on the change in skin contraction when tissue pO_2_ is raised and lowered prior to and during irradiation. The necessity for cells that determine response to reside above and below the mean pO_2_ value is not revealed by the mean tissue pO_2_. The decrease in skin contraction observed following the reduction in tissue pO_2_ by lowering the oxygen content of the breathed gas indicates that not all skin cells are fully hypoxic. Conversely, the increased skin response observed when tissue pO_2_ is elevated demonstrates that not all cells are fully oxygenated. The tissue contains cellular subpopulations existing at both higher and lower oxygen tensions, with the overall distribution yielding a mean tissue pO_2_ of approximately 15 mmHg.

The experimental findings are consistent with the distribution illustrated in Figure 6A, but not with the distribution shown in Figure 6C. The distribution in panel C contains anoxic cellular populations similar to those commonly observed in tumors [14,15].

## 4. Discussion

The increase in the skin sensitivity in mice breathing 100% oxygen and the decrease in sensitivity in mice breathing 7% and 5% oxygen versus air (20.9% oxygen) demonstrates that skin is neither fully oxygenated nor fully hypoxic. A dose of 27 Gy may be expected to deplete approximately 8–10 mmHg of intracellular oxygen [39,40,41]. As previously noted, sensitivity to radiation changes rapidly over the range of 0–10 mmHg, particularly over the 0–5 mmHg range mmHg pO_2_, with the half-maximum oxygen sensitization at ~3.5 mmHg. Over the pO_2_ range of ~15 mmHg to 760 mmHg (100% oxygen), radiation sensitivity is approximately three times greater than under very low or anoxic conditions, i.e., OER~3. Accordingly, if all cells resided in the mean tissue 15 mmHg pO_2_ environment, a FLASH tissue sparing effect would not be achieved by a dose of 27 Gy. It should be noted that the dose of radiation that deletes cellular oxygen is not dependent on the sensitivity of cells or the model which best fits the surviving fraction of cells. The surviving fraction of cells at the point when O_2_ is deleted is dependent on the cells’ radiation sensitivity. For cells residing in the sub-15 mmHg pO_2_ range, a dose of 27 Gy is sufficient to induce radiobiologic hypoxia.

As the OER relationship is independent of the response fitting model and the linear–quadratic model overestimates tissue response at doses greater than 10 Gy [43], a log-linear dose–response (**hit-target**) model was used to illustrate the impact of the different sub-15 mmHg pO_2_ distributions on cell response (Figure 6). Based on the average pO_2_ value of 15 mmHg, i.e., assuming all cells reside at the average pO_2_ value, there is essentially no difference in response to FLASH and CDR irradiation for a dose up 27 Gy, as seen in Figure 6B,D (dashed lines). However, if the pO_2_ distribution of the cells is as shown in Figure 6A, FLASH yields a significant tissue sparing effect vs. CDR irradiation (solid lines). On the other hand, the distribution in Figure 6C yields substantial but identical tissue sparing for both CDR and FLASH irradiation (solid lines) compared to all cells residing at the average of 15 mmHg pO_2_ (dashed lines). Thus, the pO_2_ distribution of cells in the 0–15 mmHg range, not the mean tissue pO_2_, governs the response of FLASH vs. CDR irradiation for doses at least up to 27 Gy. The presence of cells or tissue existing in the total absence of oxygen, as seen in Figure 6C, is more characteristic of tumors [14,15], for which a FLASH sparing effect has not been reported.

A significant finding of this study is that the percent decrease in the FLASH vs. CDR contraction in mice breathing 7% oxygen is smaller than in mice breathing 20.9% oxygen (*p* = 0.014). In mice breathing 5% oxygen, CDR contraction is minimal, ~11%, with no significant FLASH tissue sparing (*p* = 0.26). That is, in the absence or near absence of oxygen, there is no FLASH sparing effect. As seen in Figure 4, under conditions of tourniquet hypoxia, there is no FLASH sparing effect following a dose of 27 Gy or 45 Gy [34]. While the previously reported study suggests that metabolically induced hypoxia fails to yield a FLASH effect, presumably due to the absence of oxygen, the metabolic consequences of ligation and breathing low-oxygen-containing gas differ. Tourniquet ischemia not only blocks the supply of oxygen, but the sole remaining source of energy, i.e., glucose [44]. Additionally, the clearance of catabolites such as lactic acid and carbonic acid produced by the aerobic metabolism of residual tissue oxygen at the time of ligation is blocked, likely leading to intracellular acidification. The similarity in the skin contraction response to FLASH and CDR irradiation following 27 Gy irradiation under 5% oxygen breathing conditions, and tourniquet hypoxia, suggests that tourniquet-dependent metabolic changes do not abrogate the impact of oxygen deprivation. That suggests that the absence of a FLASH sparing effect following 45 Gy is also due to oxygen deprivation.

In addition to the quantitative dose response metric used in this study, i.e., skin contraction, histologic changes similarly reflect the impact of inspired oxygen concentration on skin response to radiation. Epidermal thickening is more pronounced in mice breathing oxygen, and less pronounced in mice breathing low-oxygen-content gas. In mice breathing oxygen-reduced gas and 100% oxygen, the differences between the FLASH and CDR groups did not achieve statistical significance. The histologic results reported here are consistent with other extensive studies [45], which found that, for an exposure of mouse legs to a single dose of 30 and 45 Gy, FLASH produced fewer severe toxicities and less epidermal skin thickening than CDR irradiation. RNA-seq analyses of murine skin and bone revealed pathways that were upregulated by CDR proton irradiation yet were left unaltered by FLASH irradiation, such as apoptosis signaling and keratinocyte differentiation in skin [45].

In related studies, Sesink et al. designed studies to evaluate the modifying effect of FLASH vs. CDR irradiation under air- and >90% oxygen-breathing conditions [27], and Hansen et al. evaluated skin response under air and clamp hypoxic conditions [28]. For the air and clamp hypoxia study, the endpoint was a specified level of acute skin damage up to 28 days post-irradiation. These carefully designed studies reported OER values of air breathing vs. clamp hypoxia of 1.38 for FLASH irradiation and 2.05 for CDR irradiation for the same acute skin toxicity score. The FLASH dose-modifying factor was 1.43 under normal air-breathing conditions and 0.96 under clamp hypoxic conditions. The results of those studies are complementary to the study presented here.

In the study by Sesink et al., abdominal irradiation lethality and skin toxicity were evaluated for up to 30 days post-irradiation [27]. Interestingly, the impact of altered oxygen breathing conditions appeared to differ between the skin and gastrointestinal studies, although the experiments were performed in different mouse strains. Oxygen breathing conditions did not appear to significantly affect the acute skin response.

In the study reported here for late skin contraction, a small FLASH effect under oxygen-breathing conditions was also observed, but it did not achieve statistical significance at a dose of 27 Gy. The results provide strong evidence that oxygen depletion is the mechanism of FLASH protection in mouse skin. At reduced tissue oxygenation, FLASH tissue sparing is reduced and, in the absence of oxygen, a FLASH sparing effect is not observed. This does not preclude other potential mechanisms of FLASH normal tissue sparing to play a role, for example, in other tissue, such as has been reported in the brain [46].

Montay-Gruel et al. [47] reported changes in the brains of mice exposed to FLASH and CDR irradiation, for up to 6 months following irradiation. Following 10 Gy CDR irradiation, substantial cognitive, behavioral, and morphological changes were observed, but not following FLASH irradiation. FLASH protective effect was also observed at 12 Gy, although not at 14 Gy. Interestingly, carbogen breathing eliminated FLASH sparing, suggesting a role of oxygen in the FLASH effect. The investigators further examined H_2_O_2_ production following CDR and FLASH irradiation in a cell-free system equilibrated with 4% oxygen. Upon exposure to 20–80 Gy in 10 Gy increments, the yield of H_2_O_2_ was lower by FLASH compared to CDR irradiation. However, no significant difference in H_2_O_2_ production was observed following 10 Gy FLASH and 10 Gy CDR irradiation. These results appear to substantially differ from the results of the current study in skin. As summarized by Bohlen [48], and in our previous skin studies [34], the minimum dose at which FLASH sparing becomes evident substantially exceeds 10 Gy. Moreover, the relationship between oxygen concentration and cell sensitivity is well known, and the results of the current study are quantitatively consistent with the well-established oxygen radiation sensitivity relationship. In the brain, if one assumes that FLASH tissue sparing is due to the reduced H_2_O_2_ production, then one might assume that brain sparing would be minimal at 10 Gy, a dose at which FLASH and CDR H_2_O_2_ production do not appear to differ, and become more pronounced at higher doses where the difference in H_2_O_2_ production is significant. However, such a relationship was not observed. The significant difference in the skin and brain threshold doses for a FLASH effect suggests the mechanisms of the FLASH effect in the brain and skin likely differ. A determination of the role of oxygen in FLASH brain sparing could become clearer if a reduction in brain pO_2_ reduced the magnitude of FLASH sparing.

Vaupel et al. reported median normal tissue pO_2_ values of 24 to 50 mmHg in liver, brain, skeletal muscle and gastric mucosa, with 7.5%, 10%, 8% and 0% of the recorded values in the 5–10 mmHg range, respectively [14,15]. Median pO_2_ values in normal human brain and subcutis (24 mmHg and 50 mmHg, respectively) substantially exceed the oxygen concentration that could be depleted by a clinically relevant dose of radiation. However, approximately 2% of recorded brain oxygenation values are less than 5 mmHg, and 2% of subcutaneous tissue values lie between 5 and 10 mmHg [14]. As demonstrated in [23], employing radiation sensitivity parameters of breast tissue, the presence of the 1% mildly hypoxic cells would increase the dose to yield the same level of cell survival from 20 Gy CDR to 27 Gy FLASH irradiation [23].

The long-established and well-defined quantitative relationship between pO_2_ and cell and tissue response to radiation, the presence of skin cells residing in a low pO_2_ environment, the rate of intracellular oxygen depletion in mammalian cells, and the absence of a FLASH effect in the absence of oxygen provide compelling evidence that FLASH oxygen depletion is the mechanism of normal tissue sparing in mouse skin.

## 5. Conclusions

Mean normal tissue pO_2_ values do not reveal the fraction of tissue on the threshold of hypoxia that may be rendered resistant by ultra-high dose-rate radiation at dose levels with clinical relevance. Variation in radiation-induced skin contraction in response to changes in skin oxygenation show that skin is neither fully radiobiologically hypoxic nor oxygenated. A reduction in mean tissue pO_2_ reduces skin sparing, and, under severe hypoxic conditions, the FLASH sparing effect is eliminated. An evaluation of the low-pO_2_ profiles of brain, heart and other normal tissues will be of value in determining if these tissues may exhibit radiation resistance upon exposure to FLASH irradiation.

## Data Availability

The data presented in this study are available on request from the corresponding author.

## Supplementary Materials

The following supporting information can be downloaded at: https://www.mdpi.com/article/10.3390/cancers18122011/s1, Figure S1: Mice setup, delivery parameters, and beam flatness; Figure S2: Histological alterations in mice breathing 5–100% oxygen following a single dose of 27 Gy irradiation at FLASH or CDR.

## Abbreviations

The following abbreviations are used in this manuscript:

FLASH	ultra-high dose-rate irradiation
CDR	conventional dose-rate
OER	oxygen enhancement ratio
LET	linear energy transfer
SOBP	Spread-Out Bragg Peak
H&E	Hematoxylin and Eosin
CF	contraction fraction
SF	sparing factors
DMF	dose modifying factor
