# Supplementary material for: Ultra-High Dose-Rate Oxygen Depletion and Skin Response to Irradiation
Source: Cancers (Basel). 2026 Jun 22;18(12):2011. doi: 10.3390/cancers18122011 (PMC13297236; doi:10.3390/cancers18122011)
Supplement: Supplementary file 1 [file cancers-18-02011-s001.zip › cancers-4277652-supplementary.pdf]

Supplement

A

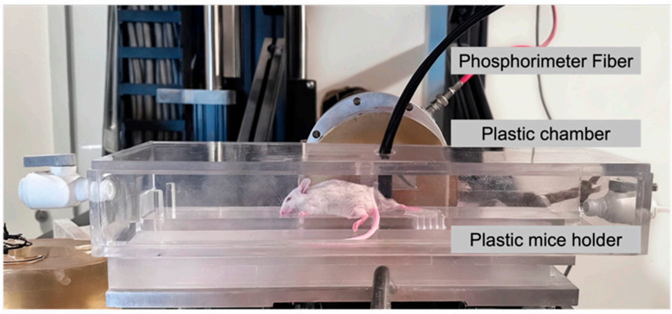

B

**Dose (Gy)**	27.02 ± 0.05
**Calibration (Gy/MU)**	2.24 ± 0.12
**FLASH (Gy/s)**	110.05 ± 6.37
**CDR (Gy/s)**	0.57 ± 0.11

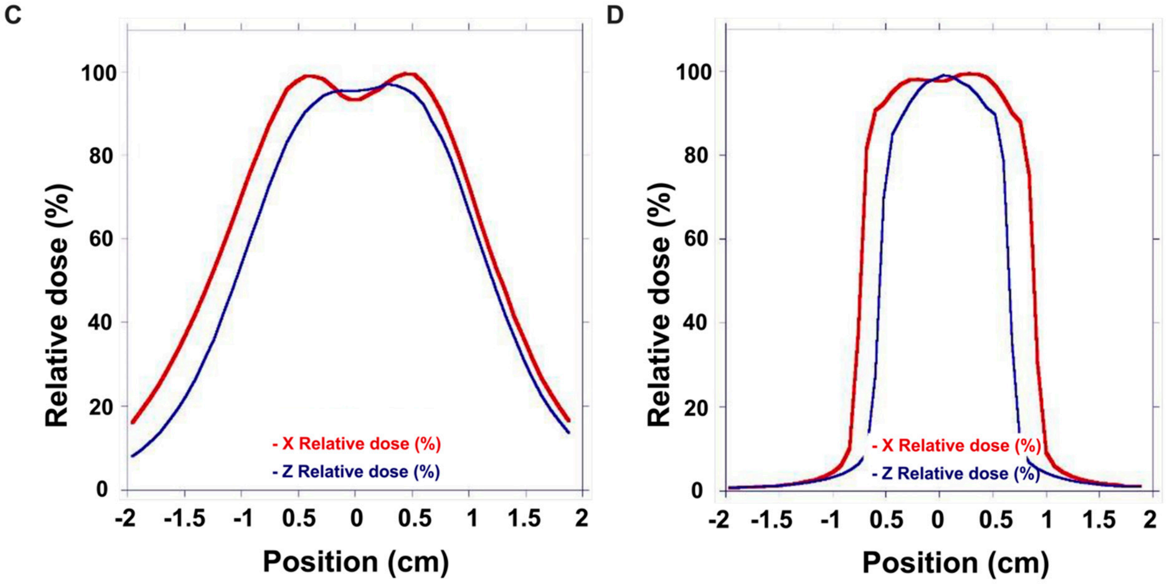

Supplementary Figure S1. **Mice setup, delivery parameters, and beam flatness.** (A) Photograph showing a mouse positioned for radiation, with phosphorimeter recording skin pO2. The beam was optimized using a double-scattering system shaped by an aperture. A polymethyl methacrylate (PMMA) block was added after the aperture to remove protons scattered in the aperture. The beam was commissioned with an extended set of dosimetry measurements (1,2). The daily dosimetry normalization was performed using a thimble chamber and a thin-gap parallel-plate ion chamber to determine the delivered Gy/MU (Gray per monitor units in the ion chamber). The mouse is restrained on a plastic custom-built platform that is placed within a custom-built plastic chamber flushed with varying oxygen levels. Small arrows indicate the direction of airflow. (B) Table displaying delivery parameters for proton irradiation, including dose, calibration (mean ± SD), FLASH and conventional dose rate (mean ± SD). A novel 2D scanning device was developed to

measure the proton beam profiles. An example of vertical and horizontal dose profiles at the target position were reconstructed and recorded without (C) and with aperture (D) in the beamline. The mice were irradiated with apertures.

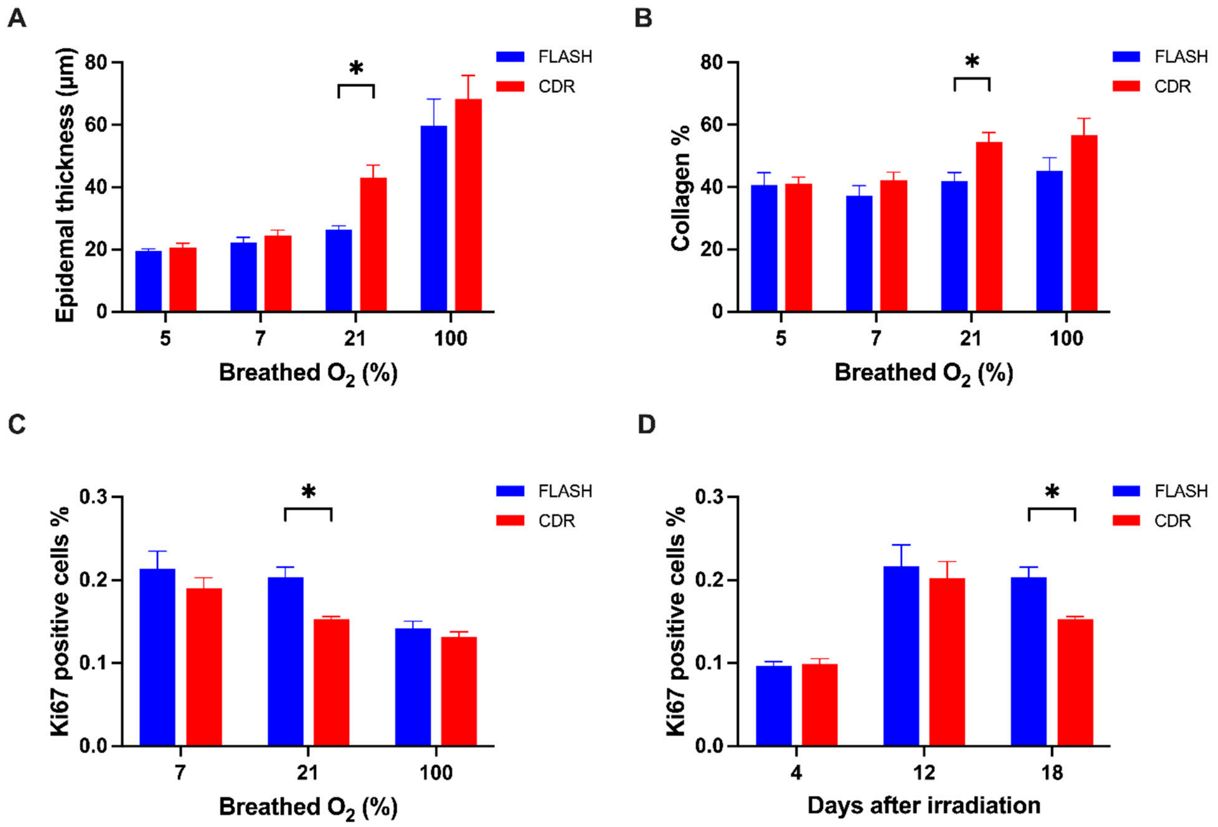

Supplementary Figure S2. **Histological alterations in mice breathing 5-100% oxygen following a single dose of 27 Gy irradiation at FLASH or CDR.** Quantification of epidermal thickness (A), collagen deposition (B), and actively proliferative cells (Ki67 positive cells) in collected skin tissues for mice breathing different oxygen concentrations 18 days after irradiations (C) and for air breathing mice after 4, 12, and 18 days post irradiation (D). N=6 for all panels, data are presented as Mean ± SEM.

Reference

1. Zhang Q, Cascio E, Li C, Yang Q, Gerweck LE, Huang P, et al. FLASH Investigations Using Protons: Design of Delivery System, Preclinical Setup and Confirmation of FLASH Effect with Protons in Animal Systems. Radiat Res. 2020 Dec 1;194(6):656–64.

2. Hachadorian R, Cascio E, Schuemann J. Increased flexibility and efficiency of a double-scattering FLASH proton beamline configuration forin vivoSOBP radiotherapy treatments. Phys Med Biol. 2023 Jul 24;68(15).
